# SEX DIFFERENCES IN LIFESPAN RESPONDING TO EARLY AND LATE ONSET DIETARY RESTRICTION

**DOI:** 10.1093/geroni/igac059.2937

**Published:** 2022-12-20

**Authors:** Nisi Jiang, Vivian Diaz, Randy Strong, James Nelson

**Affiliations:** UT Health San Antonio, San Antonio, Texas, United States; UT Health San Antonio, San Antonio, Texas, United States; UT Health San Antonio, San Antonio, Texas, United States; UT Health San Antonio, San Antonio, Texas, United States

## Abstract

Dietary restriction (DR) extends lifespan in many animal models. In mice, a range of factors affects the magnitude of the pro-longevity effect of DR, including the strain, restriction level, sex, and age of treatment onset. Early onset DR dramatically prolongs lifespan, whereas DR started later in life has diminished or no significant effect. The lifespan responses of males and females to DR also differ. Whether there is a sex difference in the lifespan response to the age of onset of DR has not been examined. Probing the basis for the variable response to DR can help elucidate the underlying mechanisms that mediate lifespan extension. We diet restricted UM-HET3 mice of both sexes from either 5 months (early-onset) or 20 months (late-onset) of age and monitored their survival. Early onset DR significantly prolonged lifespan in both males and females, which increased median lifespans by 54% and 18.5%, respectively. By contrast, although late onset DR prolonged lifespans in both sexes, the magnitude of increase in males (1.4%) was significantly lower than in females (15.8%, p=0.0474). Moreover, the decrease in the magnitude of the response to late-onset DR compared to that of early-onset DR is more dramatic in males than females. Further analysis found a significant interaction (p=0.0001) between early and late-onset DR in males but not in females (p=0.3818). Together, these results indicate that the lifespan response to delayed onset of DR diminishes more in males than in females, providing another model to probe the underlying mechanisms of DR.

